# Molecular characteristics, fitness, and virulence of high-risk and non-high-risk clones of carbapenemase-producing *Klebsiella pneumoniae*

**DOI:** 10.1128/spectrum.04036-22

**Published:** 2024-01-11

**Authors:** Anni-Maria Örmälä-Tiznado, Lisa Allander, Makaoui Maatallah, Muhammad Humaun Kabir, Sylvain Brisse, Linus Sandegren, Sheetal Patpatia, Maarten Coorens, Christian G. Giske

**Affiliations:** 1 Division of Clinical Microbiology, Department of Laboratory Medicine, Karolinska Institutet, Stockholm, Sweden; 2 Department of Medical Sciences, Uppsala University, Uppsala, Sweden; 3 Laboratoire d’Analyse, Traitement et Valorisation des Polluants de l’Environnement et des Produits (LATVPEP: LR01ES16), Faculté de Pharmacie de Monastir, Université de Monastir, Monastir, Tunisia; 4 Biodiversity and Epidemiology of Bacterial Pathogens, Institut Pasteur, Paris, France; 5 Department of Medical Biochemistry and Microbiology, Uppsala University, Uppsala, Sweden; 6 Human Microbiome Research Program, Faculty of Medicine, University of Helsinki, Helsinki, Finland; 7 Department of Clinical Microbiology, Karolinska University Hospital, Stockholm, Sweden; University of Pittsburgh School of Medicine, Pittsburgh, Pennsylvania, USA

**Keywords:** antimicrobial resistance, competition, fitness, flow cytometry, *Galleria mellonella*, *Klebsiella pneumoniae*, virulence

## Abstract

Extensively drug-resistant (XDR) *Klebsiella pneumoniae* inflict a notable burden on healthcare worldwide. Of specific concern are strains producing carbapenem-hydrolyzing enzymes, as the therapeutic options for these strains are still very limited. Specific sequence types of *K. pneumoniae* have been noted for their epidemic occurrence globally, but the mechanisms behind the success of specific clones remain unclear. Herein, we have characterized 20 high-risk clones (HiRCs) and 10 non-HiRCs of XDR *K. pneumoniae*, exploring factors connected to the epidemiological success of some clones. Isolates were subjected to core genome multilocus sequence typing analysis to determine the clonal relationships of the isolates and subsequently characterized with regard to features known to be linked to overall bacterial fitness and virulence. The genomes were analyzed *in silico* for capsule types, O antigens, virulence factors, antimicrobial resistance genes, prophages, and CRISPR-Cas loci. *In vitro* growth experiments were conducted to retrieve proxies for absolute and relative fitness for 11 HiRC and 9 non-HiRC isolates selected based on the clonal groups they belonged to, and infections in a *Galleria mellonella* insect model were used to evaluate the virulence of the isolates *in vivo*. This study did not find evidence that virulence factors, prophages, CRISPR-Cas loci, or fitness measured *in vitro* alone would contribute to the global epidemiological success of specific clones of carbapenemase-producing XDR *K. pneumoniae*. However, this study did find the HiRC group to be more virulent than the non-HiRC group when measured *in vivo* in a model with *G. mellonella*. This suggests that the virulence and epidemiological success of certain clones of *K. pneumoniae* cannot be explained by individual traits investigated in this study and thus warrant further experiments in the future.

**IMPORTANCE:**

Herein, we explored potential explanations for the successfulness of some epidemic or high-risk clones of carbapenemase-producing *Klebsiella pneumoniae*. We found differences in mortality in a larva model but found no clear genomic differences in known virulence markers. Most of the research on virulence in *K. pneumoniae* has been focused on hypervirulent strains, but here, we try to understand differences within the group of highly resistant strains. The results from the larva virulence model could be used to design experiments in higher animals. Moreover, the data could provide further support to a differentiated infection control approach against extensively drug-resistant strains, based on their classification as high-risk clones.

## INTRODUCTION

Carbapenemase-producing *Klebsiella pneumoniae* (CPKP) are a major public health concern due to the plasmid-encoded β-lactamases they carry, most notably *Klebsiella pneumoniae* carbapenemase (KPC), New Delhi metallo-β-lactamase (NDM), Verona integron-encoded metallo-β-lactamase (VIM), and oxacillinase-48 group (1). These enzymes hydrolyze most β-lactam antibiotics and thus dramatically limit the therapeutic options for infections caused by these extensively drug-resistant (XDR) *Enterobacterales* (2). *K. pneumoniae* is a natural inhabitant of the gastrointestinal tract and accounts for a variety of infections; however, the pathogenesis is multifactorial and depends upon patient factors as well as bacterial factors (3). Known *K. pneumoniae* virulence factors include capsule (K), e.g., polysaccharides, lipopolysaccharides, fimbriae, outer membrane proteins, and determinants for iron acquisition and nitrogen source utilization (4). *K. pneumoniae* infections are associated with a high rate of mortality and morbidity, in terms of both nosocomial and community-acquired infections (5). In addition, *K. pneumoniae* is a major source of resistance, most notably to carbapenems (6), and the main dissemination modes for antimicrobial resistance are the expansion of global *K. pneumoniae* clonal lineages, acquisition of multidrug-resistant (MDR) plasmids, and acquisition of antimicrobial resistance genes on transposons (7, 8).

Bacteria are continuously exposed to a variety of environmental factors and confronted with competitors and biological invaders in changing environments. Thus, the success of a given bacterial isolate is down to a complex interplay of physical and biological factors. One such factor is bacteriophages, a group of bacterial viruses that upon infection can lead to either death of the host bacterium or a symbiotic relationship, where the phage genome is integrated in the bacterial genome and continues to replicate with the host. Such integrated phage genomes are called prophages, and they can bring direct fitness advantages to their host, such as encoded toxins, increased virulence, or protection from infection by other phages. They can also act as agents of horizontal gene transfer (HGT), creating genetic variation for evolutionary innovations or bringing advantages for bacterial competition (9, 10). Clustered regularly interspaced short palindromic repeats (CRISPR) and the associated Cas-proteins provide a type of adaptive immune system for bacteria, targeting a variety of invasive genetic elements, such as viruses and plasmids (11). CRISPR-Cas systems have been suggested to play diverse roles and have the potential to modulate virulence (12), along with altering the spread of antibiotic resistance in bacterial populations by preventing the acquisition and spread of antibiotic resistance genes through HGT (13). Growth traits, such as the doubling time of bacteria, can determine how fast bacteria can exploit their host, and in many cases, the virulence of the bacterium is proportional to its rate of replication within the host (14). Furthermore, doubling time is commonly used as a measure of the fitness of bacteria (15). Competition experiments, where the ratio of two competing bacterial isolates is determined using, e.g., flow cytometry, can in turn be used to measure the relative fitness of given bacterial isolates (16).

The core genome multilocus sequence typing (cgMLST) approach is widely used for typing *K. pneumoniae*. It is based on the genome-wide comparison of 694 highly conserved genes in the core genome and the scheme cluster isolates with ≤100 allelic mismatches into clonal groups (CGs) accordingly (17). It has been shown that certain *K. pneumoniae* clones dominate the dissemination of resistance in *K. pneumoniae*, as well as being more commonly associated with epidemic spread. In addition to some hypervirulent clones, these XDR clones are referred to as global problem clones (18) or high-risk clones (HiRCs) (19). The specific mechanisms assisting the global epidemiological success of specific clones are not well known, though some factors have been suggested to play a role in the success of certain clones, such as the acquisition of acyltransferases for ST258 (20). In this study, we used the inclusion criteria for HiRC of *K. pneumoniae* as a XDR clone having caused at least four outbreaks and reported from ≥10 countries (7, 19). In this study, 30 carbapenemase-producing *K. pneumoniae* isolates were subjected to whole-genome sequencing. Isolates represented either HiRC (*n* = 20) or non-HiRC (*n* = 10) sequence types. The HiRCs considered here belong to sequence types ST11, ST14, ST17, ST147, ST512, ST258, and ST340. The non-HiRC isolates included in the study were derived from the same collections as the HiRC isolates, with no other known categorical differences apart from not meeting the criteria of being HiRC (Table 1). The isolates were characterized with regard to features known to be linked to overall bacterial fitness and virulence: *in silico* for K types, O antigens, virulence factors, antimicrobial resistance genes, prophages, and CRISPR-Cas loci. *In vitro* growth experiments were conducted to retrieve proxies for absolute and relative fitness for 11 HiRC and 9 non-HiRC isolates, and infections in a *Galleria mellonella* insect model were used to evaluate the virulence of the isolates *in vivo*. The aim of this study was to evaluate the potential differences between HiRC and non-HiRC isolates of CPKP in terms of (i) presence and prevalence of virulence factors, prophages, and CRISPR loci on the genomic level, (ii) fitness measured *in vitro,* and (iii) virulence measured *in vivo,* in order to determine whether these features could contribute to the epidemiological success of certain clones of XDR *K. pneumoniae*.

**TABLE 1:** Isolates used in the study^*a*^

Carbapenemase	Code	Isolate	ST	Country	Reference	HiRC/non-HiRC
KPC	KP1	1534*	37	USA	(21)	Non-HiRC
	KP2	10924*	334	USA	(21)	Non-HiRC
	KP3	70165	14	USA	(21)	HiRC
	KP4	70415	340	USA	(21)	HiRC
	KP5	70708*	258	USA	(21)	HiRC
	KP6	71076*	101	USA	(21)	Non-HiRC
	KP7	2008024	14	USA	(21)	HiRC
	KP8	2008025	11	USA	(21)	HiRC
	KP9	2008026*	258	USA	(21)	HiRC
	KP10	AO-8053*	512	Sweden	(22)	HiRC
VIM	KP11	AO-15200	147	Sweden	(22)	HiRC
	KP12	VPKP194*	147	Greece	(23)	HiRC
	KP13	VPKP267	147	Greece	(23)	HiRC
	KP14	VPKP205	17	Greece	(23)	HiRC
	KP15	VPKP284	676	Greece	(23)	Non-HiRC
	KP16	VPKP374*	14	Greece	(23)	HiRC
	KP17	VPKP430	14	Greece	(23)	HiRC
	KP18	VPKP389*	36	Greece	(23)	Non-HiRC
	KP19	VPKP220	147	Greece	(23)	HiRC
	KP20	VPKP229*	17	Greece	(23)	HiRC
NDM	KP21	IR11K*	14	India	(24)	HiRC
	KP22	N6*	14	India	(24)	HiRC
	KP23	IR8*	11	India	(24)	HiRC
	KP24	ED502873*	11	India	(24)	HiRC
	KP25	N12*	231	India	(24)	Non-HiRC
	KP26	N11*	147	UK	(24)	HiRC
	KP27	IR15*	38	UK	(24)	Non-HiRC
	KP28	B357*	43	UK	(24)	Non-HiRC
	KP29	IR27*	623	UK	(24)	Non-HiRC
	KP30	IR34*	624	Sweden	(24)	Non-HiRC

^*a*^ *, isolate used in the fitness and virulence experiments.

## RESULTS

### cgMLST, ICE*Kp*-associated virulence loci, K types, O antigens, and antimicrobial resistance genes

**Fig 1:**
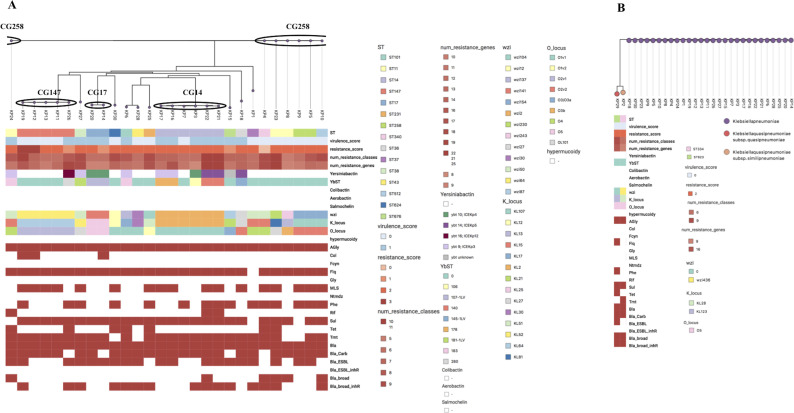
Species, ICEKp-associated virulence loci, K types, O antigens, and antimicrobial resistance genes. (A) presents isolates belonging to *K. pneumoniae,* and (B) shows the isolates belonging to other subspecies. For simplicity, the isolates in the figure are coded from KP1 to KP30; the names of the isolates are presented in Materials and Methods (Table 1). The clonal groups marked with circles represent isolates showing ≤100 allelic mismatches when analyzed with the cgMLST scheme.

According to the cgMLST scheme, the isolates clustered in four groups: CG14, CG17, CG147, and CG258. The isolates not clustering to any CG were referred to as singletons. The clonal groups consisting of isolates with ≤100 allelic mismatches are indicated in the dendrogram presented in Fig. 1. Analysis with the Kleborate tool (25–28) revealed that the isolates represented three taxa: *K. pneumoniae* (*n* = 28), *Klebsiella quasipneumoniae* subsp. *similipneumoniae* (*n* = 1), and *Klebsiella quasipneumoniae* subsp. *quasipneumoniae* (*n* = 1). The isolate K types and the O antigen (LPS) serotypes are presented in Fig. 1. No patterns were found in the distribution of K types or O antigens that would explain the differences between the HiRC and the non-HiRC isolates, and neither the virulence score nor the resistance score differed between the HiRC and the non-HiRC groups (Mann–Whitney, *P* = 0.4404 and *P* = 0.3117, respectively). The results of the Kleborate analysis, antimicrobial resistance genes, K types, and O antigens are presented in Fig. 1. In addition to the virulence genes identified in the Kleborate analysis, variants of the mannose-resistant *Klebsiella*-like type III fimbriae cluster *mrkABCDFHIJ* were present in all bacterial isolates besides VPKP220. Type III fimbrial adhesin is necessary for biofilm formation on abiotic surfaces and surfaces coated with host-derived extracellular matrix proteins. It has been suggested that type III fimbriae are important in biofilm-mediated infections on indwelling devices, including catheter-associated urinary tract infections (29). Moreover, VPKP284 (ST676, K locus KL21) was found to contain *allD*, *allR, arcC,* and *glxR* genes connected to anaerobic assimilation of allantoin, which have been associated with hypervirulent *K. pneumoniae* strains that cause pyogenic liver abscesses (29).

### CRISPR loci

CRISPR loci along with accompanying *cas*-genes were found in 13 isolates. An orphan *cas*-locus without the CRISPR region was found in one isolate, and isolated CRISPR loci without cas-genes were found in three isolates (Table 2). The presence of a complete CRISPR-Cas system did not differ between the HiRC and the non-HiRC groups (Mann–Whitney, *P* = 0.8177). However, the presence of complete CRISPR-Cas regions was found to be different between isolates belonging to different clonal groups (Kruskal–Wallis, *P* = 0.0044). The number of CRISPR loci, along with the cas-genes, is presented in Table 2.

**TABLE 2:** CRISPR-Cas loci

Clonal group	Isolate	No. of CRISPR loci	Cas	Complete CRISPR-Cas
CC258	70415	0	0	No
	70708	0	0	No
	2008025	0	0	No
	2008026	0	0	No
	AO-8053	0	0	No
	IR8	0	0	No
	ED502873	0	0	No
CC147	AO-15200	2	TypelE	Yes
	VPKP194	2	TypelE	Yes
	VPKP267	2	TypelE	Yes
	VPKP220E	2	TypeIE, TypeIV	Yes
	N11	1	TypelE	Yes
CC14	70165	2	TypelE	Yes
	2008024	2	TypelE	Yes
	VPKP374	2	0	No
	VPKP430	2	0	No
	IR11K	2	TypelE	Yes
	N6	2	TypelE	Yes
CC17	VPKP205	0	0	No
	VPKP2290	0	0	No
Singleton	1534	0	0	No
	10924	0	TypelV	No
	71076	0	0	No
	VPKP284	0	0	No
	VPKP389	1	TypelE	Yes
	N12	1	TypelV	Yes
	IR15	1	TypelV	Yes
	B357	1	0	No
	IR27	0	0	No
	IR34	1	TypelV	Yes

### Prophages

All of the isolates (*n* = 30) were found to contain prophages, and the number of prophages in the individual bacterial genomes ranged from 3 to 13. The number of prophages did not differ between HiRC vs. non-HiRC clones (Mann–Whitney, *P* = 0.3117) or between the clonal groups (ANOVA, *P* = 0.6928), and any specific CDs were not found to be overrepresented in either of the groups. The list of prophages, along with the phage genome length, position within the bacterial genome, whether the phage is intact or incomplete, the CG% content of the phage genome, and whether the genes found in the prophages matched bacterial or virus databases are presented in detail in Table S1. When looking at the percentage of genes of bacterial origin that the prophages were carrying, there was no difference between the HiRC and the non-HiRC groups (unpaired *t*-test, *P* = 0.4459).

### Absolute fitness measured as doubling time

**Fig 2:**
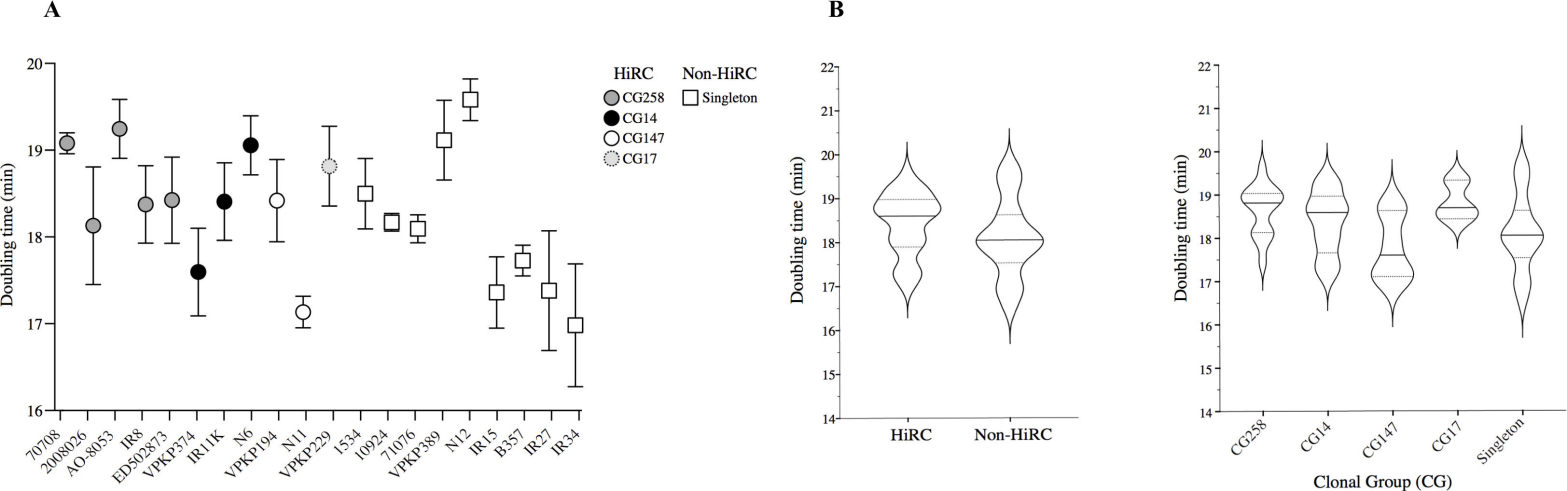
(**A**) Doubling time (min) calculated as log(2)/slope at exponential phase, representing growth rate of the bacterial isolates. Error bars denote the standard deviation (SD) of three biological replicates. (**B**) Doubling time (min), representing growth rate of the bacterial isolates grouped by HiRC isolates vs non-HiRC isolates and clonal groups. Three biological replicates per isolate included. The median doubling time is illustrated by the filled line, and the dashed line denotes interquartile range.

The doubling time (min) for HiRC (*n* = 11) and non-HiRC (*n* = 9) isolates was measured by optical density at OD = 600 nm, in order to evaluate potential differences in the absolute fitness. The doubling time is obtained by calculating log(2)/slope at the exponential growth phase and illustrated for each isolate in Fig. 2A. There was no significant difference in doubling time between the HiRC and non-HiRC groups (unpaired *t*-test, *P* = 0.1238) or among clonal groups (ANOVA, *P* = 0.0807). The doubling time of isolates grouped as HiRC or non-HiRC and by the clonal group is illustrated in Fig. 2B.

### Competition experiments (relative fitness)

**Fig 3:**
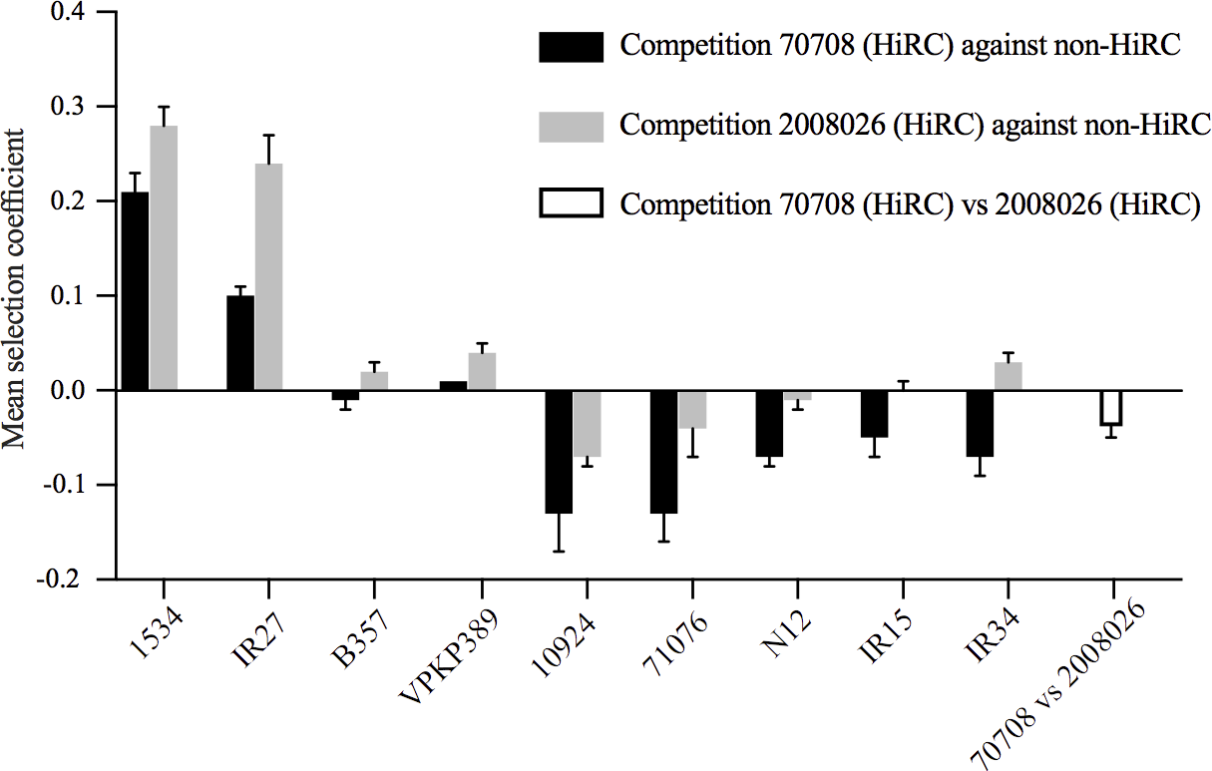
Relative fitness (mean selection coefficient) of fluorescently marked HiRC isolates 70708-*yfp* and 2008026-*yfp* to unmarked non-HiRC isolates, measured by competition experiments. The mean selection coefficient was compensated for the cost of YFP in 70708 and 2008026. Error bars denote the SD.

In order to study relative fitness, competition experiments between HiRC and non-HiRC isolates were performed. 70708YFP (CG258) and 2006026YFP (CG258) were tagged by inserting genes expressing fluorescent protein (YFP) and competed against nine unmarked non-HiRC isolates (Table 1). The selection coefficient (*s*-value), calculated as the difference in the ratio between isolates per generation, represents a measure of relative fitness between the competing isolates. There was a difference in selection coefficients between the isolates (ANOVA, *P* < 0.0001). The relative fitness between the non-HiRC isolates and the HiRC isolates 70708 (CG258) and 2008026 (CG258), respectively, is presented in Fig. 3. Six out of nine non-HiRC isolates had a fitness advantage against the HiRC isolate 70708, and three out of nine non-HiRC isolates had a fitness advantage against the HiRC isolate 2008026. The HiRC isolate 2008026 outcompeted non-HiRC isolates more often than 70708.

### Virulence experiments with *Galleria mellonella* larvae

#### Survival

**Fig 4:**
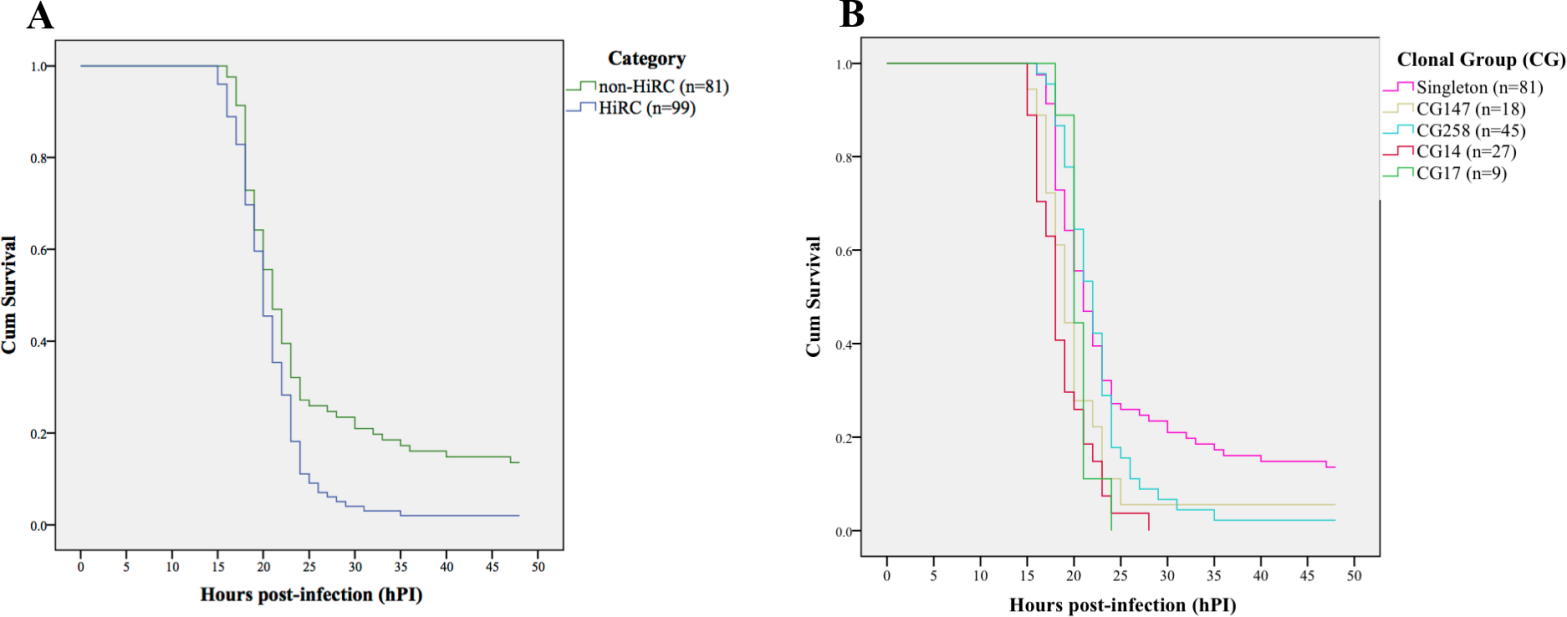
Kaplan–Meier survival curve illustrating the survival of 180 *Galleria mellonella* larvae. The larva survival distribution is grouped by (A) HiRC vs non-HiRC isolates and (B) clonal groups of the bacteria used in the infections.

**Fig 5:**
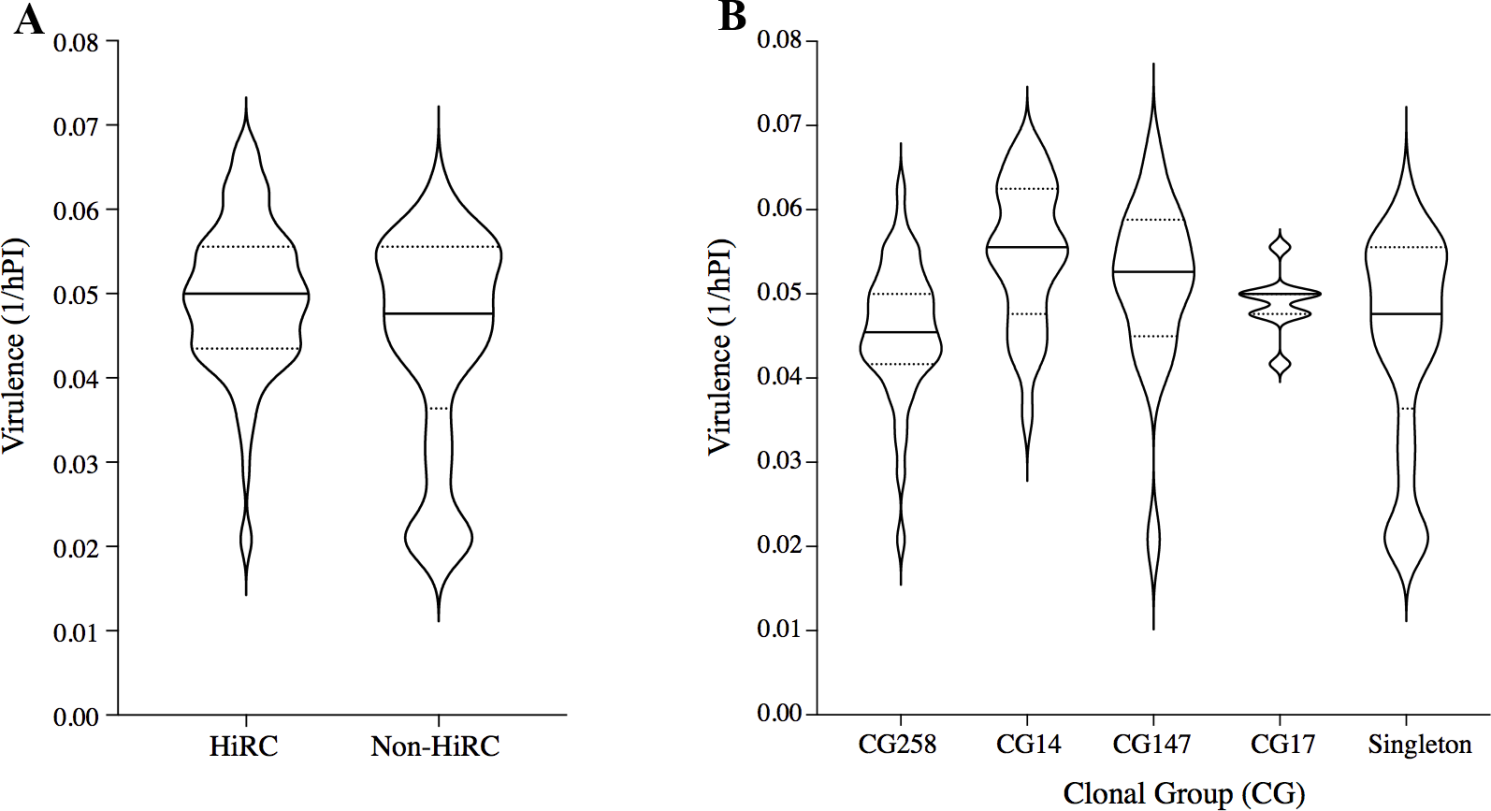
Virulence (1/hPI) of the isolates grouped by (A) HiRC (*n* = 11) vs non-HiRC (*n* = 9) isolates and (B) clonal group (CG258, *n* = 5; CG14, *n* = 6; CG147, *n* = 2; CG17, *n* = 1; and singleton, *n* = 9). Nine larvae per isolate were included. The median doubling time is illustrated by the filled line, and the dashed line denotes interquartile range. As controls, larvae were injected with H_2_O or did not receive any injection at all (Neg CTRL).

The *G. mellonella* larva infection model was used for assessing virulence of a set of HiRC (*n* = 11) and non-HiRC (*n* = 9) isolates. Kaplan–Meier survival analysis was used to plot the survival distribution of larvae (Fig. 5). There was a difference in the larva survival times when the survival distribution was grouped by infections with HiRC vs non-HiRC isolates (log rank: χ^2^ = 9.636, df = 1, *P* = 0.002; Breslow: χ^2^ = 4.498, df = 1, *P* = 0.034) (Fig. 4). Most larvae died between 15 and 25 hours post-infection in both groups. The median survival times for larvae infected with HiRC isolates were 20 hours, whereas the median survival time for larvae infected with non-HiRC isolates was 21 hours. In general, more larvae survived the infection with the non-HiRC isolates; after the 48-hour monitoring, 12.2% of the larvae infected with non-HiRC isolates were still alive, in contrast to 2.2% of the larvae infected with HiRC isolates. There was also a difference in survival time depending on which clonal group the isolate belonged to (log rank test: χ^2^ = 25.521, df = 4, *P* < 0.0001; Breslow: χ^2^ = 28.387, df = 4, *P* < 0.0001). The Kaplan–Meier survival curve grouped by the clonal group of the bacteria used for the infection is presented in Fig. 4.

#### Virulence

The virulence of a given isolate was defined as 1/(time of death in hours post-infection), i.e., 1/hPI. There was a significant difference in virulence (1/hPI) between the HiRC and non-HiRC groups (Mann–Whitney *U* = 3,273, *n*_1_ = 99, *n*_2_ = 81, *P* = 0.0332), illustrated in Fig. 5A. Furthermore, there was a difference in virulence between the clonal groups [Kruskal–Wallis (χ^2^ = 22.21, *P* = 0.0002)]; CG14 was shown to have a higher mean virulence level compared to CG258 (*P* = 0.001) and singleton (*P* = 0.001) (Fig. 5B).

### Correlation between doubling time and virulence

There was a negative correlation between doubling time and virulence in the non-HiRC group (*r*_s_ = −0.481, *P* = 0.011); however, this was not observed for the HiRC group (*P* = 0.128).

## DISCUSSION

The 30 KPC-, NDM-, or VIM-producing XDR *K. pneumoniae* used in this study fell into four different genetic clusters based on the cgMLST typing scheme and were assigned to clonal groups CG14, CG17, CG147, and CG258 accordingly. The rest of the isolates were assigned as singletons, considered as non-HiRCs. The clusters were not associated with geography or the type of carbapenemase that the isolates produced, as isolates representing different countries and carbapenemases were found within all clonal groups, with the exception of CG17 consisting of only two isolates, both VIM producers from Greece.

The virulence observed in the *G. mellonella* model was found to be higher in the group consisting of HiRC isolates as compared to the non-HiRC isolates, thus further validating the grouping into alleged HiRC vs non-HiRCs. The higher mean virulence observed in the HiRC group was not predicted by the *in silico* analysis of the virulence factors. When increasing the resolution and comparing the clonal groups to one another, there was notable variation within the HiRC and non-HiRC groups, suggesting that the observed differences in virulence could arise rather on the clonal group level, further emphasizing the complexity of the factors and strategies contributing to epidemiological success.

Capsule types and other virulence factors have an undisputable role in the pathogenesis and survival of the bacteria, but the prevalence or distribution of virulence factors in our data set did not indicate that these factors would be explanatory of why some clonal lineages are more often associated with global epidemics. A new putative virulence gene was identified in VPKP220 showing similarity to type III fimbriae cluster gene *mrkD,* along with the genes connected to the anaerobic utilization of allantoin as a nitrogen source found in VPKP284 (*allD, allR, arcC, and glxR*), which are most often present in isolates causing liver abscesses (30–32). The ICEKp-associated virulence loci, K types, O antigens, and antimicrobial resistance gene profiles showed similarities within the clonal groups; however, evidence of specific features explaining why some clones pose a higher risk in terms of epidemic spread was not found.

Notably, all of the *K. pneumoniae* isolates in the study carried a significant load of prophages, and the number of prophages within an isolate varied from 3 to 13. The overall prophage diversity in *K. pneumoniae* is not well documented to date, and there are only a few recent studies on the topic (33), and thus, this finding warrants future research. The PHASTER tool (34) identifies the genes found in the prophage regions to be hits either against virus and prophage database or bacterial database. The percentage of genes that matched the bacterial database were calculated for each phage region, as genes of bacterial origin could potentially be beneficial for the bacteria. However, the percentage of genes of bacterial origin within the prophage regions was not found to differ between the HiRC and non-HiRC groups. Noteworthy, individual genes conferring a substantial selection advantage could overshadow such quantitative differences, should they exist between the two groups.

Overall, the *in silico* analyses did not reveal factors potentially contributing to the success of international high-risk clones, with one exception. The presence of CRISPR-Cas systems was found to be different among clonal groups, and interestingly, the clones belonging to CG258 did not harbor any complete CRISPR-Cas systems or isolated CRISPR or cas loci. As opposed to the hypervirulent clones of *K. pneumoniae*, the resistant clones are highly diverse with frequent chromosomal recombination and gene content variability (35), and the evolution of resistance is largely driven through the acquisition of antimicrobial resistance genes on diverse mobile genetic elements (MGEs) (19). These MGEs are particularly prevalent among the subset of globally disseminated high-risk clones, such as CG258 (36), and the complete lack of CRISPR-Cas systems in CG258 could have allowed a greater uptake of foreign genetic material. This finding is further supported by a study by Mackow et al., showing that CRISPR-Cas was absent in most clinically important ST258 clones (37). When two isolates belonging to CG258 (70708 and 2008026) were competed in a liquid culture against a number of non-HiRC isolates belonging to the singleton group, they had a relative fitness disadvantage against some isolates and no or very low fitness advantage over other isolates. This suggests that the HiRC isolates belonging to CG258 are not successful due to a competition advantage in the absence of antimicrobial pressure, at least *in vitro*. This, however, is likely to change substantially in the presence of antimicrobial selection.

Interestingly, there was a negative correlation between doubling time and virulence in the non-HiRC group, but this was not true for the HiRC isolates. The virulence being correlated with growth ability can often indicate that the virulence could be at least partially coupled with the doubling time, through, for instance, more effective or rapid colonization of the host (14). Virulence being correlated with growth only in the non-HiRC group suggests a difference in the general life history strategies between the HiRC and non-HiRC groups. More specifically, the virulence of the non-HiRCs is likely to be connected to their growth ability, while HiRCs possess other characteristics enabling them to be virulent independent of their growth characteristics.

This study did not find evidence that virulence factors, prophages, CRISPR-Cas loci, or fitness measured *in vitro* alone would contribute to the global epidemiological success of specific clones of carbapenemase-producing XDR *K. pneumoniae*. However, this study did show differences between the HiRC and non-HiRC groups in terms of virulence measured *in vivo*. In order to understand the mechanisms behind the observed differences and, ultimately, how to harness this knowledge into infection prevention and control, further in-depth studies are needed, not the least in the presence of antimicrobials. Moreover, the observed differences between the clonal groups further emphasize the complexity of the life history strategies, suggesting that the global epidemiological success of XDR *K. pneumoniae* is likely to arise from multiple differing strategies intertwining from a variety of bacterial traits. Lastly, the results from the *in vivo* experiments call for further experiments in more complex animal models or cell lines, as the differences in virulence between the HiRC and non-HiRC groups found in this study could not simply be explained by genomic features or fitness measures *in vitro*.

## MATERIALS AND METHODS

### Bacterial strains and cultures

The inclusion criteria for HiRC of *K. pneumoniae* as a MDR clone having caused at least four outbreaks and reported from ≥10 countries were used (7, 19). The isolates used in the study were (i) sent to the CDC for reference antimicrobial susceptibility testing with limited clinical information on the specimen type (KP1–KP9) (21), (ii) isolated from expectorate, urine, perineal swab, blood culture, and wound secretions (KP10, KP11) (22), (iii) isolated from urine, sputum, wound, blood, catheter, and pus samples (KP12–KP20) (23), or (iv) isolated from urinary and lower respiratory tract samples (KP21–KP30) (24).

The bacteria were stored at −80°C and cultivated on cystine-lactose-electrolyte-deficient (CLED) or Mueller–Hinton II (MHII) (Sigma Aldrich) agar plates. Liquid cultures were done in MHII broth (Sigma Aldrich) or Luria–Bertani (LB) broth (Sigma Aldrich) unless stated otherwise. The isolates used in this study are presented in Table 1. Additional strains used for the construction of strains with fluorescent markers are listed in Table S2.

### Whole-genome sequencing and assembly

All isolates (*n* = 30) were subjected to whole-genome sequencing. After overnight aerobic incubation on MHII agar at 37°C, two to five pure colonies were taken with inoculation then suspended in Mueller–Hinton Broth (pH 7.4). The cell suspensions were aliquoted in a 96-well plate, and genomic DNA was extracted using an automated DNA extraction system (MagNA Pure 96, Roche Life Sciences, Sweden). DNA concentrations were measured with Qubit 3.0 (Thermo Scientific, MA, USA). Sequencing libraries were generated using Nextera XT (Illumina kits), and short-read sequencing was run on Illumina (HiSeq 2500) systems with a 150 bp insert size paired-end sequencing protocol at Science for Life Laboratory, Stockholm, Sweden. Sequences were assembled with SPAdes (v. 3.10.0).

### Core genome multilocus sequence typing

The *K. pneumoniae* isolates (*n* = 30) were characterized by cgMLST. Sequences were uploaded to the bacterial isolate genome sequence database (BIGSdb-Kp) (https://bigsdb.pasteur.fr/klebsiella/) to scan sequences and define clonal groups with isolate showing ≤100 allelic mismatches with one another. Clonal groups were defined by the curator of the database.

### Capsule types, virulence factors, CRISPR-Cas, and prophages

The Kleborate tool (https://github.com/katholt/Kleborate) (25–28) was used to identify species, ICEKp-associated virulence loci, antimicrobial resistance genes, K types, and O antigens. Additional virulence genes were identified using the BIGSdb‐Kp database (https://bigsdb.pasteur.fr/klebsiella/), and screening for CRISPR-Cas loci was done with CRISPR-Cas++ (https://crisprcas.i2bc.paris-saclay.fr). For the identification of the phage regions, the online tool PHAST was used (http://phaster.ca) (38).

### Bacterial growth rate

Bacterial growth was measured as optical density (OD_600_) using the Bioscreen C MBR spectrophotometer (Oy Growth Curves Ab Ltd). One microliter overnight culture (~1 **×** 10^9^ CFU/mL in MHII broth, Sigma Aldrich) was added to 2 mL MHII media, whereafter 300 µL was transferred to 100-well “Honeycomb 2” plates (Oy Growth Curves Ab Ltd). Three biological replicates were included, as well as one technical replicate for each biological replicate. Bacterial growth was measured at 37°C with OD measurements every 4 min for 24 h. Plotting of bacterial growth curves and calculation of doubling time were performed using BAT 2.0 (Bioscreen Analysis Tool) (39). BAT 2.0 is a web browser version of an R-script (R Foundation for Statistical Computing) developed for analyzing growth curves from Bioscreen C MBR machines. For OD values between 0.02 and 0.09, bacterial growth was found to be exponential, and the doubling time (min) was calculated by log(2)/slope of the curve during the exponential growth phase.

### Infection model with *Galleria mellonella* larvae

*G. mellonella* larvae were acquired from Vivara Naturprodukter (Gothenburg, Sweden) and kept at room temperature in darkness with food. Larvae were used in the infection experiments 1 day after arrival. Larvae that did not show any gray pigmentation and were responsive to physical stimuli were defined as healthy and used in the experiments. Bacterial virulence was measured by infecting *G. mellonella larvae*. For each isolate, three biological replicates were used, and three larvae per biological replicate was infected, giving a total of nine infected larvae per isolate. Bacteria were grown in MHII broth overnight, and larvae were injected with 10 µL bacterial suspension, containing approximately 1 × 10^9^ CFU/mL, in the last right pro-leg using a U-100 insulin syringe [0.5 mL, 8 mm (5/16″) × 29 ga]. In the same manner, nine larvae were injected with 10 µL dH_2_O as a control to account for physical trauma caused by the injection. A control group of nine larvae that did not receive any injection was additionally included. The larvae were kept at room temperature, and larvae mortality was recorded at 1-hour intervals for 48 hours. The death of larvae was characterized by a lack of response to physical stimuli (by, for example, gently shaking the Petri dish). The time of death (hours post-infection) was used in Kaplan–Meier survival analysis in order to compute and compare survival over time.

#### Competition assay with multicolor flow cytometry (MACSquant)

Competition experiments were performed between two fluorescently marked (YFP) HirC isolates and unmarked non-HirC isolates. The construction of strains with fluorescent markers is described in Method S1, along with the PCR cycles used in the construction (Method S2). Six biological replicates were included. Overnight cultures (1 × 10^9^ CFU/mL in LB media) were mixed 1:1 in 2 mL LB (1,000-fold dilution) and passaged with a 1,000-fold dilution following an overnight incubation at 37°C. The ratio between the competing bacteria was measured every ~24 hours using multicolor flow cytometry (MACSquant). The two ratios were fitted to the function, where *y* is the ratio, *G* is a constant, *s* is the selection coefficient, and *t* is the time in generations. The calculation was performed using CompDAta software (version 4.4) (available from https://pikkeiyuen.se/app/). Single outliers that likely represent media adaptation mutants were removed if diverging greatly from the normal distribution of remaining replicates. The mean selection coefficient was compensated for the cost of YFP in 70708 and 2008026.

#### Statistical analyses

Statistical analyses were performed in GraphPad Prism 9 (GraphPad Software, LLC, version 9.0.2). An assessment of the normality of data was performed with Kolmogorov–Smirnov (K-S) and Shapiro–Wilk tests, as well as through analysis of normal *QQ* plots. Levene’s test for equality of variance and analysis of homoscedasticity plots were used to assess the homogeneity of variance across different groups. Differences between two groups for parametric data were analyzed with an unpaired *t*-test, and differences among more than two groups were assessed using one-way ANOVA. Multiple pairwise comparisons of parametric data were performed with GLM *post hoc* tests. The *post hoc* test used was Tukey’s honestly significant difference (HSD) and least significant difference (LSD) (assuming equal variance) or Games–Howell, Tamhane’s T2, and Dunnett’s T3 when the assumption of homogeneity of variance was violated (adjusts for unequal variance). For non-parametric data, differences between two groups were analyzed with the Mann–Whitney *U* test, and differences among more than two groups were analyzed with the Kruskal–Wallis test (one-way analysis of variance on ranks) and involving multiple pairwise comparisons in the test. The survival data retrieved from the *G. mellonella* infection model was assessed with Kaplan–Meier survival analysis (i.e., calculation of survival distribution of infected larvae). The differences between group survival distributions were evaluated with the log rank test and Breslow test (tests for comparison of survival distributions). Two-tailed bivariate Spearman rho correlation analysis was performed to evaluate the correlation and strength of correlation between doubling time and virulence.

## Data Availability

The *K. pneumoniae* genomes were deposited in the NCBI GenBank database under the following accession numbers: KP1 (JAWZYR000000000), KP2 (JAWZYQ000000000), KP3 (JAWZYP000000000), KP4 (JAWZYO000000000), KP5 (JAWZYN000000000), KP6 (JAWZYM000000000), KP7 (JAWZYL000000000), KP8 (JAWZYK000000000), KP9 (JAWZYJ000000000), KP10 (JAWZYI000000000), KP11 (JAWZYH000000000), KP12 (JAWZYG000000000), KP13 (JAWZYF000000000), KP14 (JAWZYE000000000), KP15 (JAWZYD000000000), KP16 (JAWZYC000000000), KP17 (JAWZYB000000000), KP18 (JAWZYA000000000), KP19 (JAWZXZ000000000), KP20 (JAWZXY000000000), KP21 (JAWZXX000000000), KP22 (JAWZXW000000000), KP23 (JAWZXV000000000), KP24 (JAWZXU000000000), KP25 (JAWZXT000000000), KP26 (JAWZXS000000000), KP27 (JAWZXR000000000), KP28 (JAWZXQ000000000), KP29 (JAWZXP000000000), and KP30 (JAWZXO000000000).

## SUPPLEMENTAL MATERIAL

Method S1 spectrum.04036-22-s0001.docx Construction of strains with fluorescent markers.

Method S2 spectrum.04036-22-s0002.docx PCR cycles and primers.

Table S1 spectrum.04036-22-s0003.pdf Prophages.

Table S2 spectrum.04036-22-s0004.pdf Strains used for construction of strains with fluorescent markers.

ASM does not own the copyrights to Supplemental Material that may be linked to, or accessed through, an article. The authors have granted ASM a non-exclusive, world-wide license to publish the Supplemental Material files. Please contact the corresponding author directly for reuse.
