# Supplementary material for: Molecular characteristics, fitness, and virulence of high-risk and non-high-risk clones of carbapenemase-producing Klebsiella pneumoniae
Source: Microbiol Spectr. 2024 Jan 11;12(2):e04036-22. doi: 10.1128/spectrum.04036-22 (PMC10845972; doi:10.1128/spectrum.04036-22)
Supplement: Method S1 — Construction of strains with fluorescent markers. [file spectrum.04036-22-s0001.docx]

Method S1. Construction of strains with fluorescent markers

*Preparation of electrocompetent cells and transformation with pSIM5-Tet plasmid*

The pSIM5-Tet plasmid (DA24100) encoding λ-Red recombination components was purified using the E.Z.N.A Plasmid DNA Mini Kit I (Omega Bio-Tek^TM^, VWR). 500 μl of overnight culture (~1 x 10^9^ CFU/ml in LB media) of selected *K. pneumoniae* isolates were inoculated in 45 ml LB media and grown at 37°C to an OD_600_ ≈ 0.5-0.6. The cultures were subsequently transferred to ice for 10 min and continuously swirled to ensure even cooling. The cultures were transferred to ice-cold 50 ml falcon tubes and centrifuged 15 min at 4500 rpm at 4°C. The cell pellet was resuspended in 40 ml, 25 ml and 10 ml ice-cold 10% glycerol respectively and centrifuged 15 min at 4500 rpm at 4°C between each wash. The cell pellet was resuspended in 2 ml ice-cold 10% glycerol and centrifuged at 10000 x g at 4°C for 20 min. Lastly, the cell pellet was resuspended in 500 μl ice-cold 10% glycerol. The cells were continuously kept on ice. 50 μl of electrocompetent cells was mixed with 1 μl (~100ng) of pSIM5-Tet plasmid DNA and transferred to a precooled electroporation cuvette. Electroporation was performed by using Gene Pulser Xcell system (Bio-Rad™) set at 2.5 kV, 25 μF and 200 Ω. Negative controls were included by electroporation of cells without presence of plasmid DNA. The electroporated cells were immediately diluted with 1 ml of warm Brain heart infusion (BHI) growth medium (Sigma Aldrich) and incubated at 30°C overnight. Following overnight incubation, transformants were selected by plaiting 1:1, 1:10, 1:100 dilutions on MH II agar plates containing tetracycline (12.5 μg/ml). The plates were incubated at 30°C overnight. Colonies were re-streaked on MH II agar plates containing tetracycline (12.5 μg/ml) to confirm successful transformation with pSIM5-Tet plasmid.

*Preparation of electrocompetent cells for λ-red based transformation*

500 μl of overnight cultures (1 x 10^9^ CFU/ml in LB) of *K. pneumoniae/*pSIM5-Tet transformants were inoculated in 45ml of LB and grown at 30°C to an OD_600_ = 0.2 - 0.3. The flasks were thereafter placed in a 42°C shaking water bath for 15min to induce of λ-red recombination functions. The cultures were put on ice for 10 min and repeatedly swirled. The cooled culture was centrifuged at 4500 rpm at 4°C for 15 min. The cell pellets were resuspended in 20 ml, 15 ml, 10 ml, ice-cold 10% glycerol, respectively, and centrifuged for 15 min at 4500 rpm at 4°C following every wash. The cell pellet was resuspended in 2 ml ice-cold 10% glycerol and centrifuged at 10000 x g at 4°C for 20 min. Lastly, the cell pellet was resuspended in 500 μl ice-cold 10% glycerol. The cells were continuously kept on ice.

*λ-red based integration of Zeo-sacB cassette*

50 μl of electrocompetent *K. pneumoniae*/pSIM5 cells were mixed with ~200ng de-salted PCR product of Zeocin-*sacB* cassette (Thermo Scientific™ GeneJET™ Gel Extraction Kit) (see Supplemental material S4) and transferred to a precooled electroporation cuvette. Electroporation was performed by using Gene Pulser Xcell system (Bio-Rad™) set at 2.5 kV, 25 μF and 200 Ω. Negative controls were included by electroporation cells without adding *zeo-sacB* cassette. The electroporated cells were immediately diluted with 1 ml of warm SOC medium (Super Optimal broth with Catabolite repression, Sigma Aldrich) and put in a 42°C water bath for 15 min to boost λ-red recombination functions. The cultures were henceforth incubated with shaking at 30°C for 3 h. Transformants were selected for by plating 1:1, 1:10, 1:100 dilutions on low-salt LA plates (Sigma Aldrich) containing Zeocin (25 μg/ml). The plates were incubated at 30°C overnight. Transformants were re-streaking on zeocin plates (25 μg/ml), patched on LA + 5% sucrose plates. Maintenance of pSIM5 plasmid in transformants was verified by plating on MH-II agar with tetracycline (12.5 μg/ml). Successful integration of *zeo-sacB* cassette in *galK* was verified using PCR. The primers and PCR program used are described in Supplementary material S4.

*λ-red based integration of fluorescent marker gene in K. pneumoniae galK::zeo-sacB/pSIM5*

50 μl of electrocompetent *K. pneumoniae* *galK::zeo-sacB*/pSIM5 was mixed with ~200ng de-salted PCR product of  *yfp,* (see Supplementary material S4) and transferred to a precooled electroporation cuvette. Electroporation was performed by using Gene Pulser Xcell system (Bio-Rad™) set at 2.5 kV, 25 μF and 200 Ω. Negative controls were included by electroporation cells without fluorescence marker DNA. The electroporated cells were immediately diluted with 1 ml of warm SOC media (Sigma Aldrich) and put in 42^o^C water bath for 15 min to boost λ-red recombination functions. 4 ml LB was added and the cultures were incubated with shaking at 37^o^C overnight. Transformants were counter-selected by plaiting 1:1, 1:10, 1:100 dilutions on LA + 5% sucrose. The plates were incubated 37^o^C overnight. Successful exchange of the *zeo-sacB* cassette for fluorescence gene was verified by checking for fluorescence and with PCR screening of *galK*. Removal of pSIM5-tet plasmid was verified by patching on MH-II agar with tetracycline (12.5 μg/ml).
