# Supplementary material for: Molecular characteristics, fitness, and virulence of high-risk and non-high-risk clones of carbapenemase-producing Klebsiella pneumoniae
Source: Microbiol Spectr. 2024 Jan 11;12(2):e04036-22. doi: 10.1128/spectrum.04036-22 (PMC10845972; doi:10.1128/spectrum.04036-22)
Supplement: Method S2 — PCR cycles and primers. [file spectrum.04036-22-s0002.docx]

Method S2. PCR cycles and primers

*Amplification of Zeocin-sacB cassette*

Amplification of Zeocin-sacB (DA33713) was performed using Thermo Scientific™ Phusion™ High-Fidelity DNA Polymerase kit according to the manufacturer’s instructions

using primers 5´- TTGGCAACGGCGTGCTGAACGGTATCGACGAGATCTTCGGTGTAGGCTGGAGCTGCTTC -3´ and 3´- CGCGGTCAGCGACGTCGATTTTCGTGAATCGGGAGTGTAACATATGAATATCGTCGTTAGTTCG -5’. The PCR amplification cycle consisted of 5 minutes at 98°C, followed by 2 cycles of 30 seconds at 98°C, 30 seconds at 57°C and 1 minute 56 seconds in 72 °C, two cycles of 30 seconds at 98°C, 30 seconds at 55°C and 1 minute 56 seconds at 72°C, 9 cycles of 30 seconds at 98°C, 30 seconds at 51°C and 1 minute 56 seconds in 72 °C, 22 cycles of 30 seconds at 98°C and 1 minute 56 seconds in 72 °C.

*Screening for galK*

Screening for *galk* was performed using primers 5´-TACGGGTTTTCGCTTCATACT-3´ and 5´-GTTATGAGATGCTGGCGGAA-3´ with Thermo Scientific™ DreamTaq PCR kit according to manufacturer’s instructions. The DNA template was prepared by suspending one bacterial colony in 100 μl dH_2_O. The PCR amplification cycle for amplification of *galK* consisted of 5 minutes at 94°C, followed by 30 cycles of 30 seconds at 94°C, 30 seconds at 55°C and 2 minutes 40 seconds at 72°C, followed by a single extension time of 7 minutes at 72°C.

*Amplification of fluorescent marker genes*

Amplifications of the fluorescence gene *yfp* (DA55343) was performed using primers 5´-TTGGCAACGGCGTGCTGAACGGTATCGACGAGATCTTCGGATCAAGGAGAAGAAACAAACTT-3´ and 5´-CGCGGTCAGCGACGTCGATTTTCGTGAATCGGGAGTGTAATAAGATCGCTGCGATTTG-3´ and Thermo Scientific™ Phusion™ High-Fidelity DNA Polymerase kit according to manufacturer’s instructions. The DNA template was prepared by suspending 1 μl culture kept at -80°C in 50 μl H_2_O. The PCR amplification cycle for amplification of *yfp* consisted of 5 minutes at 98°C, 2 cycles of 30 seconds at 98°C,  30 seconds at 57°C and 48 seconds at 72°C, 2 cycles of 30 seconds at 98°C,  30 seconds at 54°C and 48 seconds at 72°C, 9 cycles of 30 seconds at 98°C,  30 seconds at 51°C and 48 seconds at 72°C, 22 cycles of 30 seconds at 98°C and 48 seconds at 72°C, and finally a single extension time of 7 minutes at 72°C
