# Supplementary material for: Molecular characteristics, fitness, and virulence of high-risk and non-high-risk clones of carbapenemase-producing Klebsiella pneumoniae
Source: Microbiol Spectr. 2024 Jan 11;12(2):e04036-22. doi: 10.1128/spectrum.04036-22 (PMC10845972; doi:10.1128/spectrum.04036-22)
Supplement: Table S1 — Prophages. [file spectrum.04036-22-s0003.pdf]

Table S1. Prophages

Clonal group	ST	Isolate	No. prophage regions	Possible phage	Intact	Position	No. CDs	Amount of phage DNA (kb)	CG %	Hits against Virus and prophage DB	Hits against Bacterial DB or GenBank file	% of bacterial genes
	11	2008025	11	PHAGE_Escher_HK639_NC_016158	Y	296572-349576	51	53	52.94%			
				PHAGE_Entero_Fels_2_NC_010463	Y	948470-975548	35	27	52.23%			
				PHAGE_Salmon_RE_2010_NC_019488	Y	1639399-1679041	47	39,6	52.76%			
				PHAGE_Entero_phiV10_NC_007804	Q	2023153-2048582	24	25,4	54.16%			
				PHAGE_Salmon_ST64B_NC_004313	Q	3207084-3239730	26	32,6	52.96%			
				PHAGE_Thermu_phiYS40_NC_008584	N	3894565-3909240	15	14,6	57.92%			
				PHAGE_Salmon_Fels_1_NC_010391	Y	4227450-4286058	64	58,6	53.82%			
				PHAGE_Acinet_Bphi_B1251_NC_019541	Q	4334277-4349129	21	14,8	52.53%			
				PHAGE_Cronob_ENT47670_NC_019927	Y	4400974-4464280	79	63,3	53.17%			
				PHAGE_Entero_P4_NC_001609	Q	4532276-4567947	15	35,6	54.35%			
				PHAGE_Entero_P4_NC_001609	Q	4557069-4578159	24	21	53.90%			
							401	385,5		299	102	25,44
	11	IR8	6	PHAGE_Haemop_Aaphi23_NC_004827	Y	431041-472166	55	41,1	51.94%			
				PHAGE_Cronob_ENT47670_NC_019927	Y	3053602-3098876	60	45,2	53.18%			
				PHAGE_Salmon_RE_2010_NC_019488	Y	3191831-3216561	30	24,7	56.55%			
				PHAGE_Salmon_ST64B_NC_004313	Q	3481564-3510072	27	28,5	52.60%			
				PHAGE_Entero_Fels_2_NC_010463	Y	4568162-4587458	25	19,2	51.08%			
				PHAGE_Entero_P4_NC_001609	Y	5289148-5302911	22	13,7	55.72%			
							219	172,4		158	61	27,8
	11	ED502873	6	PHAGE_Haemop_Aaphi23_NC_004827	Y	558280-618421	56	60,1	53.90%			
				PHAGE_Salmon_RE_2010_NC_019488	Y	956509-990783	42	34,2	51.00%			
				PHAGE_Salmon_SSU5_JQ965645	Y	2213849-2328622	118	114,7	49.18%			
				PHAGE_Salmon_ST64B_NC_004313	Q	3913178-3943581	26	30,4	52.94%			
				PHAGE_Cronob_ENT47670_NC_019927	Y	4860291-4914791	76	54,5	53.01%			
				PHAGE_Entero_P4_NC_001609	N	5175743-5179941	6	4,1	53.18%			
							324	298		237	87	26,85
	258	70708	7	PHAGE_Salmon_Fels_1_NC_010391		253-28654	39	28.4	51.71%			
				PHAGE_Entero_Fels_2_NC_010463		1213567-1250033	50	36.4	51.16%			
				PHAGE_Entero_phiV10_NC_007804		1330546-1380926	47	50.3	54.18%			
				PHAGE_Salmon_RE_2010_NC_019488		2384015-2423657	46	39.6	52.76%			
				PHAGE_Salmon_ST64B_NC_004313		4333146-4366068	26	32.9	52.95%			
				PHAGE_Entero_P4_NC_001609		4980799-4996846	20	16	58.30%			
**CC258**				PHAGE_Sphing_PAU_NC_019521		5345274-5376852	26	31.5	49.03%			
							235.1			187	67	26,38
	258	2008026	7	PHAGE_Entero_phiV10_NC_007804	Q	987427-1038157	45	50.7	54.84%			
				PHAGE_Salmon_Fels_1_NC_010391	Q	1493910-1526976	29	33	51.93%			
				PHAGE_Entero_Fels_2_NC_010463	Y	2180100-2219742	45	39.6	52.76%			
				PHAGE_Entero_Fels_2_NC_010463	Y	2516663-2549988	44	33.3	51.46%			
				PHAGE_Salmon_ST64B_NC_004313	Q	3732025-3760274	26	28.2	52.59%			
				PHAGE_Stx2_converting_1717_NC_011357	N	4461168-4479889	23	18.7	40.77%			
				PHAGE_Entero_P4_NC_001609	Y	5290783-5303960	23	13.1	54.23%			
							235	216.6		172	63	26,81
	512	AO-8053	10	PHAGE_Entero_phiV10_NC_007804	Y	1259944-1310184	47	50.2	53.03%			
				PHAGE_Entero_Fels_2_NC_010463	Y	1580076-1615583	47	35.5	51.17%			
				PHAGE_Salmon_RE_2010_NC_019488	Y	2583467-2623109	45	39.6	52.76%			
				PHAGE_Salmon_Fels_1_NC_010391	Q	3500040-3546581	39	46.5	54.71%			
				PHAGE_Salmon_ST64B_NC_004313	Q	4828687-4859090	26	30.4	52.93%			
				PHAGE_Cronob_ENT47670_NC_019927	Q	5102014-5150590	63	48.5	54.05%			
				PHAGE_Escher_HK639_NC_016158	N	5336476-5354044	26	17.5	52.95%			
				PHAGE_Acinet_Bphi_B1251_NC_019541.	Y	5359260-5403451	33	44.1	52.25%			
				PHAGE_Haemop_Aaphi23_NC_004827	Q	5425958-5452826	43	26.8	48.25%			
				PHAGE_Entero_P4_NC_001609	N	5616736-5626723	16	9.9	55.02%			
							385	349		275	110	28,57
	340	70415	12	PHAGE_Haemop_Aaphi23_NC_004827	Y	525106-581248	56	56.1	53.34%			
				PHAGE_Salmon_ST64B_NC_004313	Q	4244277-4274680	26	30.4	52.94%			
				PHAGE_Pandor_inopinatum_NC_026440	N	4517727-4532687	16	14.9	59.77%			
				PHAGE_Stx2_converting_1717_NC_011357	N	4697087-4703533	11	6.4	52.35%			
				PHAGE_Entero_BP_4795_NC_004813	Y	5143695-5186086	24	42.3	48.16%			
				PHAGE_Entero_Fels_2_NC_010463	Y	5291271-5318808	35	27.5	54.50%			
				PHAGE_Escher_Av_05_NC_025830	N	5357900-5389938	34	32	47.59%			
				PHAGE_Entero_Fels_2_NC_010463	Y	5388205-5430140	68	41.9	53.02%			
				PHAGE_Entero_P1_NC_005856	N	5481548-5512307	21	30.7	49.54%			
				PHAGE_Erwini_ENT90_NC_019932	Y	5575236-5592249	28	17	51.45%			
				PHAGE_Salmon_RE_2010_NC_019488	Y	5609799-5690709	107	80.9	52.29%			
				PHAGE_Salmon_RE_2010_NC_019488	N	5683668-5689267	10	5.6	54.30%			
								385.7		240	196	44,95
	147	AO-15200	6	PHAGE_Entero_mEp237_NC_019704	N	1693502-1724533	26	31	53.53%			
				PHAGE_Entero_PsP3_NC_005340	Y	3229471-3270844	50	41.3	51.90%			
				PHAGE_Salmon_SSU5_JQ965645	Y	3414818-3566505	159	151.6	49.84%			
				PHAGE_Acinet_Bphi_B1251_NC_019541	N	4601945-4613864	18	11.9	51.38%			
				PHAGE_Pseudo_phiPSA1_NC_024365	Q	4790385-4811707	24	21.3	58.68%			
				PHAGE_Klebsi_phiKO2_NC_005857	Y	5318575-5428269	113	109.6	51.69%			
								366.7		321	69	17,7
	147	VPKP194	6	PHAGE_Thermu_phiYS40_NC_008584	N	727756-742808	16	15	60.11%			
				PHAGE_Entero_mEp237_NC_019704	Y	839735-905225	48	65,4	53.13%			
				PHAGE_Entero_PsP3_NC_005340	Y	1383986-1421817	45	37,8	51.41%			
				PHAGE_Salmon_SSU5_JQ965645	Y	4545771-4659132	117	113,3	49.35%			
				PHAGE_Salmon_SSU5_NC_018843	N	5238302-5249204	21	10,9	45.50%			
				PHAGE_Entero_mEp235_NC_019708	Y	5304107-5353859	46	49,7	52.73%			
							293	292,1		236	57	19,45
	147	VPKP267	5	PHAGE_Entero_mEp237_NC_019704	Y	846581-912534	49	65.9Kb	52.87%			
				PHAGE_Pseudo_vB_PaeS_PMG1_NC_016765	Y	2494394-2537871	43	43.4Kb	51.23%			
				PHAGE_Entero_PsP3_NC_005340	Y	2852621-2890452	42	37.8Kb	51.41%			
				PHAGE_Salmon_SSU5_JQ965645	Y	3571791-3688757	110	116.9Kb	49.53%			
**CC147**				PHAGE_Prochl_P_HM2_NC_015284	N	3994047-4006473	10	12.4Kb	58.97%			
								276.4		212	42	16,54
	147	VPKP220	7	PHAGE_Entero_mEp237_NC_019704		261-55303	47	55	53.06%			
				PHAGE_Salmon_SSU5_JQ965645		3453562-3556356	104	102,7	49.38%			
				PHAGE_Entero_PsP3_NC_005340		3932532-3973905	50	41,3	51.90%			
				PHAGE_Stx2_converting_1717_NC_011357		4277181-4300213	28	23	55.09%			
				PHAGE_Entero_mEp235_NC_019708		4663532-4684973	25	21,4	52.69%			
				PHAGE_Entero_P1_NC_005856		4780652-4805499	22	24,8	51.22%			
				PHAGE_Entero_mEp390_NC_019721		4912048-4925124	22	13	49.22%			
							298	281,2		242	56	18,79
	147	N11	9	PHAGE_Entero_mEp237_NC_019704		2326874-2382160	55	55.2	51.34%			
				PHAGE_Entero_PsP3_NC_005340		2650176-2691549	49	41.3	51.89%			
				PHAGE_Salmon_SSU5_NC_018843		3757740-3771884	24	14.1	54.14%			
				PHAGE_Salmon_SSU5_NC_018843		3929123-3939291	13	10.1	52.34%			
				PHAGE_Salmon_SSU5_JQ965645		3937131-4005906	86	68.7	51.17%			

				PHAGE_Salmon_SSU5_NC_018843		4303759-4326094	25	22.3	51.35%			
				PHAGE_Escher_Av_05_NC_025830		5142140-5173721	18	31.5	49.87%			
				PHAGE_Entero_mEp235_NC_019708		5178892-5217416	46	38.5	51.70%			
				PHAGE_Entero_N15_NC_001901		5684781-5776677	114	91.8	50.09%			
							430	373.5		317	113	26,28
	17	VPKP205	5	PHAGE_Entero_P88_NC_026014	N	1502861-1527116	27	24.2	53.46%			
				PHAGE_Entero_PsP3_NC_005340	Y	1906391-1942305	44	35.9	52.17%			
				PHAGE_Salmon_vB_SemP_Emek_NC_018275	Q	3502056-3527461	25	25.4	47.62%			
				PHAGE_Pectob_ZF40_NC_019522	Y	3819659-3865475	44	45.8	51.04%			
				PHAGE_Entero_P4_NC_001609	Q	4328366-4375253	30	46.8	52.12%			
				PHAGE_Stx2_converting_1717_NC_011357			170	178.1		115	461	80,03
**CC17**	17	VPKP229	8	PHAGE_Entero_phiV10_NC_007804	Q	568-40761	48	40.1	54.40%			
				PHAGE_Pectob_ZF40_NC_019522	Q	130498-161230	38	30.7	51.31%			
				PHAGE_Salmon_SSU5_JQ965645	Y	2022940-2132661	121	109.7	49.77%			
				PHAGE_Entero_P88_NC_026014	N	2175057-2213264	42	38.2	50.13%			
				PHAGE_Entero_HK544_NC_019767	Y	2382958-2438170	49	55.2	51.59%			
				PHAGE_Salmon_vB_SosS_Oslo_NC_018279	N	3233549-3268245	29	34.6	54.18%			
				PHAGE_Entero_P4_NC_001609	Q	3302508-3331942	30	29.4	52.06%			
				PHAGE_Entero_PsP3_NC_005340	Y	3952826-3992327	49	39.5	52.61%			
							406	281.2		321	85	20,96
	14	2008024	6	PHAGE_Salmon_SPN1S_NC_016761	Q	857802-902664	45	44.8	52.72%			
				PHAGE_Salmon_SSU5_JQ965645	Y	2657606-2772086	99	114.4	48.89%			
				PHAGE_Entero_P4_NC_001609	Q	3686573-3718427	16	31.8	50.01%			
				PHAGE_Entero_HK140_NC_019710	Y	3736109-3770644	39	34.5	51.21%			
				PHAGE_Entero_mEp237_NC_019704	Y	4269734-4342749	78	73	53.80%			
				PHAGE_Cronob_ENT47670_NC_019927	Y	5246676-5283430	50	36.7	52.41%			
							327	335.2		259	68	20,8
	14	VPKP374	6	PHAGE_Entero_P88_NC_026014	N	1999012-2035218	40	36.2Kb	50.75%			
				PHAGE_Entero_P4_NC_001609	Q	3632249-3657326	17	25Kb	49.01%			
				PHAGE_Entero_HK022_NC_002166	N	3652046-3685509	31	33.4Kb	48.59%			
				PHAGE_Cronob_ENT47670_NC_019927	Y	3696534-3749330	63	52.7Kb	51.96%			
				PHAGE_Cronob_ENT47670_NC_019927	N	4178100-4195832	26	17.7Kb	55.06%			
				PHAGE_Entero_HK140_NC_019710	Y	5394348-5438272	49	43.9Kb	52.57%			
								208.9		158	69	30,4
	14	VPKP430	13	PHAGE_Salmon_SSU5_JQ965645	Y	6917-113337	109	106.4	49.46%			
				PHAGE_Pectob_ZF40_NC_019522	Q	331421-367607	32	36.1	52.31%			
				PHAGE_Haemop_Aaphi23_NC_004827	Q	391797-435072	62	43.2	53.27%			
				PHAGE_Entero_P88_NC_026014	N	1852801-1868713	22	15.9	56.50%			
				PHAGE_Entero_P4_NC_001609	Q	2124119-2135928	12	11.8	50.03%			
				PHAGE_Entero_mEp237_NC_019704	Y	3503268-3551469	55	48.2	52.06%			
				PHAGE_Entero_c_1_NC_019706	Y	5384005-5414486	43	30.4	55.41%			
				PHAGE_Entero_P4_NC_001609	N	5514286-5528065	17	13.7	50.64%			
				PHAGE_Entero_N15_NC_001901	N	5623300-5648119	28	24.8	49.09%			
				PHAGE_Shigel_SfIV_NC_022749	N	5696413-5706978	12	10.5	54.80%			
				PHAGE_Escher_HK639_NC_016158	N	5819814-5832782	19	12.9	50.45%			
				PHAGE_Entero_mEp390_NC_019721	Q	5838306-5860449	16	22.1	52.68%			
				PHAGE_Klebsi_phiKO2_NC_005857	Y	5885358-5926586	48	41.2	52.78%			
							475	377.2		373	102	21,47
**CC14**												
	14	IR11K	8	PHAGE_Entero_P4_NC_001609	N	535976-556040	15	20	51.93%			
				PHAGE_Salmon_E1_NC_010495	Y	3267377-3311296	60	43.9	53.11%			
				PHAGE_Entero_P88_NC_026014	N	3441374-3477580	40	36.2	50.76%			
				PHAGE_Entero_Fels_2_NC_010463	Y	3521997-3547879	32	25.8	54.55%			
				PHAGE_Entero_mEp237_NC_019704	Q	4701572-4772800	75	71.2	54.97%			
				PHAGE_Entero_HK140_NC_019710	Y	4822785-4870575	39	47.7	51.46%			
				PHAGE_Escher_HK639_NC_016158	Y	4890352-4938054	63	47.7	51.13%			
				PHAGE_Escher_HK639_NC_016158	N	5194543-5203932	14	9.3	52.76%			
							338	301,8		242	82	25,31
	14	N6	6	PHAGE_Entero_phiP27_NC_003356	N	882411-902475	15	20	51.93%			
				PHAGE_Vibrio_pYD38_A_NC_021534	Y	1721336-1780825	59	59.4	53.40%			
				PHAGE_Entero_P88_NC_026014	N	2012372-2048578	38	36.2	50.76%			
				PHAGE_Entero_Fels_2_NC_010463	Y	3095579-3121461	33	25.8	54.55%			
				PHAGE_Entero_HK140_NC_019710	Y	4064600-4088123	31	23.5	52.61%			
				PHAGE_Stx2_converting_1717_NC_011357	N	4875939-4896544	24	20.6	58.28%			
							200	301.8		143	57	28,5
	14	70165	10	PHAGE_Salmon_SPN1S_NC_016761	Q	1567765-1612627	47	44.8	52.72%			
				PHAGE_Salmon_SSU5_JQ965645	Y	2742077-2854003	101	111.9	48.83%			
				PHAGE_Entero_mEp237_NC_019704	N	4166750-4195939	39	29.1	49.20%			
				PHAGE_Entero_P4_NC_001609	Q	4217561-4249300	18	31.7	50.00%			
				PHAGE_Entero_HK544_NC_019767	Y	4705167-4727977	27	22.8	52.17%			
				PHAGE_Cronob_ENT47670_NC_019927	Y	5103111-5139220	50	36.1	52.43%			
				PHAGE_Entero_HK140_NC_019710	Y	5179574-5194397	23	14.8	50.95%			
				PHAGE_Shigel_SfIV_NC_022749	Y	5215284-5252639	27	37.3	54.82%			
				PHAGE_Shigel_SfIV_NC_022749	N	5264252-5273681	14	9.4	54.64%			
				PHAGE_Escher_HK639_NC_016158	Q	5279720-5308152	29	28.4	53.46%			
							375	366.3		303	72	19,2
	36	VPKP389	10	PHAGE_Entero_P88_NC_026014	N	1599202-1635408	38	36.2	50.75%			
				PHAGE_Salmon_SSU5_JQ965645	Y	3301694-3415753	105	114	49.21%			
				PHAGE_Entero_SfI_NC_027339	N	3934804-3950063	30	15.2	52.44%			
				PHAGE_Vibrio_pYD38_A_NC_021534	Q	4078113-4094445	26	16.3	52.39%			
				PHAGE_Entero_P4_NC_001609	Q	4096716-4124755	19	28	49.85%			
				PHAGE_Entero_HK140_NC_019710	Y	4134421-4171105	32	36.6	55.92%			
				PHAGE_Entero_P1_NC_005856	N	4169328-4215762	25	46.4	51.11%			
				PHAGE_Pseudo_73_NC_007806	N	4255487-4267350	13	11.8	52.20%			
				PHAGE_Ectoca_siliculosus_virus_1_NC_002687	Y	4806747-4823719	21	16.9	50.93%			
				PHAGE_Entero_mEp390_NC_019721	N	5348022-5370440	23	22.4	50.40%			
							332	343.8		242	90	27,11
	37	1534	10	PHAGE_Salmon_vB_SosS_Oslo_NC_018279	Y	2931639-2992425	52	60.7	54.50%			
				PHAGE_Salmon_Fels_1_NC_010391	N	3761339-3803731	28	42.3	53.03%			
				PHAGE_Pseudo_D3_NC_002484	N	3825818-3839369	12	13.5	50.86%			
				PHAGE_Shigel_SfII_NC_021857	Y	4613162-4655566	44	42.4	54.48%			
				PHAGE_Entero_mEp390_NC_019721	Q	5442034-5462190	34	20.1	55.50%			
				PHAGE_Entero_HK140_NC_019710	N	5648439-5668368	14	19.9	52.95%			
				PHAGE_Shigel_SfIV_NC_022749	Y	5671618-5713729	27	42.1	50.76%			
				PHAGE_Entero_epsilon15_NC_004775	Q	5709667-5717492	14	7.8	52.38%			
							260	296.7		184	76	29,23
	38	IR15	4	PHAGE_Entero_mEp237_NC_019704		2040782-2068407	34	27.6	51.31%			
				PHAGE_Entero_SfI_NC_027339		2538376-2586756	66	48.3	51.86%			
				PHAGE_Entero_PsP3_NC_005340		3140404-3179327	43	38.9	52.80%			
				PHAGE_Escher_HK639_NC_016158		4990202-5014734	27	24.5	52.24%			
							170	139.3		136	34	20
	43	B357	8	PHAGE_Salmon_ST64B_NC_004313	Q	565939-582396	17	16.4	49.91%			
				PHAGE_Entero_P88_NC_026014 .	N	2810095-2845189	40	35K	51.14%			

				PHAGE_Thermu_phiYS40_NC_008584 .	N	2847905-2865543	14	17.6	56.85%			
				PHAGE_Entero_mEp390_NC_019721	N	3092718-3123767	18	31	54.51%			
				PHAGE_Entero_PsP3_NC_005340	Y	3132473-3167691	47	35.2	54.46%			
				PHAGE_Entero_P4_NC_001609	Q	3755610-3768531	15	12.9	50.20%			
				PHAGE_Entero_P1_NC_005856	N	4269997-4303445	35	33.4	54.52%			
				PHAGE_Entero_SfV_NC_003444	Y	4342846-4369257	45	26.4	50.02%			
							231	207.9		154	77	33,33
	101	71076	13	PHAGE_Entero_Fels_2_NC_010463	Y	472210-508959	45	36.7	50.44%			
				PHAGE_Salmon_ST64B_NC_004313	Y	1327024-1382513	46	55.4	47.75%			
**Singleton**				PHAGE_Escher_TL_2011b_NC_019445	Y	1665948-1710982	60	45	52.48%			
				PHAGE_Salmon_vB_SosS_Oslo_NC_018279	N	1714249-1732009	18	17.7	51.44%			
				PHAGE_Klebsi_phiKO2_NC_005857	Q	3314217-3357206	33	42.9	50.66%			
				PHAGE_Entero_P88_NC_026014	N	3351839-3393962	39	42.1	55.23%			
				PHAGE_Entero_P4_NC_001609	N	3723761-3756543	13	32.7	57.62%			
				PHAGE_Klebsi_phiKO2_NC_005857	N	4000217-4026700	23	26.4	47.60%			
				PHAGE_Klebsi_phiKO2_NC_005857	Y	4064910-4105451	46	40.5	51.08%			
				PHAGE_Entero_Sf6_NC_005344	N	4469590-4476093	10	6.5	54.52%			
				PHAGE_Klebsi_phiKO2_NC_005857	N	4864068-4885573	30	21.5	50.72%			
				PHAGE_Cronob_ENT47670_NC_019927	Y	5166151-5215955	61	49.8	52.79%			
				PHAGE_Burkho_BcepMu_NC_005882	Y	5744126-5776395	46	32.2	56.08%			
							470	449.4		364	106	22,55
	334	10924	4	PHAGE_Entero_N15_NC_001901	Y	1189614-1231693	46	42	52.69%			
				PHAGE_Vibrio_pYD38_A_NC_021534	Q	3012554-3056000	47	43.4	56.25%			
				PHAGE_Entero_c_1_NC_019706	Y	3190784-3217805	29	27	50.71%			
				PHAGE_Entero_P88_NC_026014	Y	5071007-5110186	41	39.1	53.80%	136	27	16,56
								151.5				
	623	IR27	6	PHAGE_Salmon_SPN1S_NC_016761	Y	562133-613333	52	51.2	53.88%			
				PHAGE_Entero_P4_NC_001609	N	1823282-1859493	17	36.2	48.83%			
				PHAGE_Entero_Fels_2_NC_010463	Y	3460447-3495658	40	35.2	52.57%			
				PHAGE_Bacill_Bobb_NC_024792	N	4045202-4052660	9	7.4	45.34%			
				PHAGE_Entero_mEp237_NC_019704	Y	4184934-4257869	58	72.9	52.97%			
				PHAGE_Shigel_SfII_NC_021857	Y	4671451-4726478	53	55	51.47%			
								257.9		174	55	24,02
	624	IR34	3	PHAGE_Entero_mEp237_NC_019704	Q	431502-467666	34	36.1	51.24%			
				PHAGE_Vibrio_pYD38_A_NC_021534	Y	3780724-3863172	75	82.4	53.40%			
				PHAGE_Entero_P4_NC_001609	Q	4026150-4047110	13	20.9	53.56%			
								139.4		90	32	26,23
	676	VPKP284	4	PHAGE_Entero_mEp237_NC_019704	N	1799210-1836551	32	37.3	54.32%			
				PHAGE_Acinet_Bphi_B1251_NC_019541	Q	4632507-4654039	20	21.5	53.50%			
				PHAGE_Klebsi_phiKO2_NC_005857	Q	4762162-4813789	66	51.6	51.78%			
				PHAGE_Klebsi_phiKO2_NC_005857	Q	5452026-5497962	25	45.9	54.22%			
							143	156.3		127	16	11,19
	231	N12	4	PHAGE_Entero_P4_NC_001609	N	1222926-1233565	12	10.6	46.46%			
				PHAGE_Entero_mEp213_NC_019720	Y	2201625-2244678	48	43	49.47%			
				PHAGE_Psychr_pOW20_A_NC_020841	Y	2493970-2550688	80	56.7	54.53%			
				PHAGE_Escher_Av_05_NC_025830	N	5220799-5231160	27	10.3	45.56%			
							167	120,6		117	50	29,94
