# Supplementary material for: Molecular characteristics, fitness, and virulence of high-risk and non-high-risk clones of carbapenemase-producing Klebsiella pneumoniae
Source: Microbiol Spectr. 2024 Jan 11;12(2):e04036-22. doi: 10.1128/spectrum.04036-22 (PMC10845972; doi:10.1128/spectrum.04036-22)
Supplement: Table S2 — Strains used for construction of strains with fluorescent markers. [file spectrum.04036-22-s0004.pdf]

Table S2. Strains used for the construction of strains with fluorescent markers

Strain		Purpose
DA24100	*E. coli* pSIM5-Tet	Source of plasmid carrying the λ-red recombination components
DA33713	*S. typhimurium cobA::ble-sacB*-T0	Template for amplification of zeocin-sacB cassette
DA55343	*E. coli ΔIS150::CP25-SYFP2*	Template for amplification of fluorescent marker gene (*yfp*) with homology to *galK* in *Klebsiella pneumoniae*
